# Prevalence of eating disorders in patients with celiac disease: a comparative study with healthy individuals

**DOI:** 10.1590/1806-9282.20231090

**Published:** 2024-03-15

**Authors:** Renato Nisihara, Ana Clara Maier Techy, Carolina Staichok, Thais Carolini Roth, Grácia Furiatti de Biassio, Luani Risso Cardoso, Lorete Maria da Silva Kotze

**Affiliations:** 1 Universidade Positivo, Department of Medicine – Curitiba (PR), Brazil.; 2 Universidade Federal do Paraná, Clinical Hospital – Curitiba (PR), Brazil.

**Keywords:** Celiac disease, Eating disorders, Anorexia, Bulimia, Binge eating disorder, Depression

## Abstract

**INTRODUCTION:**

Celiac disease is a chronic immune-mediated disease, which is triggered and maintained by gluten in genetically susceptible individuals. Eating disorders are a persistent disturbance in eating-related behavior that results in altered food consumption or absorption and that significantly impairs physical health or psychosocial functioning.

**OBJECTIVE:**

This study aimed at evaluating the prevalence of eating disorders in Brazilian celiac patients.

**METHODS:**

This cross-sectional study was conducted as online survey including adult celiac patients who agreed to participate and a paired control health group. Questionnaires included questions about socioeconomic data and celiac disease diagnosis, and a validated questionnaire about eating disorders (Eating Attitudes Test-26.

**RESULTS:**

In total, 741 responses were studied, with 484 from the celiac group and 257 from the control group. No significant difference was observed between the number of individuals at risk of developing eating disorder (p=0.39). Both groups showed a high risk of developing eating disorders (34.2% in the celiac group and 37.7% in the control group). Furthermore, among patients with celiac disease, we found higher scores on the Eating Attitudes Test-26 in those with depression (p=0.0013), those with living difficulty due to the disease (p<0.0001), and those dissatisfied with their weight (p<0.0001).

**CONCLUSION:**

In the sample analyzed, no greater risk of eating disorders was identified in patients with celiac disease compared with the control group. However, in general, about one-third of the respondents in each group had scores associated with the risk of eating disorders. Among celiac patients, depression, difficulties living with celiac disease, and being unhappy with one's weight were associated with higher risk for eating disorder.

## INTRODUCTION

Celiac disease (CD) is a chronic, systemic, and immune-mediated disease that happens from exposure to gluten present in food, mainly affecting the small intestine and generating gastrointestinal and non-gastrointestinal symptoms^1^. Due to the accessibility of the diagnosis, its incidence has increased globally^2,3^. The treatment consists of a gluten-free diet (GFD), which improves the symptoms, avoiding complications^4^. Both symptomatology and dietary restriction negatively interfere with the patients’ quality of life. Some authors have reported that much longer the time since diagnosis and GFD is, the more adapted the patients are^3^.

Eating disorders (EDs) are a persistent disturbance in eating or eating-related behavior that results in altered food consumption and that significantly impairs physical health or psychosocial functioning^5^. Among these disorders, anorexia nervosa (AN), bulimia nervosa (BN), and binge ED (BED) are classified as specific EDs. These diseases are characterized by continuous disturbances in eating, different weight control modalities, and exacerbated care with aesthetics/body weight^6^. According to the WHO, the estimated prevalence of ED is 4.7% in the general Brazilian population^7^. Is it possible that the need to restrict the diet of celiac people is capable of generating ED? Studies carried out in the United States, Sweden, and Poland showed a higher frequency of ED in celiac patients^8^. In the Spanish population, there is no significant difference in the prevalence of ED between celiac patients and people in general^9^.

People with CD must strictly avoid gluten-containing foods to manage their condition effectively. This restrictive diet can lead to a heightened focus on food, eating habits, and body weight. In some cases, this can manifest as disordered eating patterns, such as binge eating, purging, or extreme dietary restrictions, which are the characteristics of various EDs^8,9^. There is a genetic association between the immunoregulatory mechanisms of CD and common metabolic pathways for diabetes and AN, favoring the coexistence of these chronic diseases^10^. Additionally, there was a similarity in the epidemiological profile of celiac patients with those with ED, the majority of whom were women, Caucasian, and young^11^.

In Brazil, no studies were found on the frequency of ED among patients with CD. Thus, we aimed to investigate the frequency of ED in celiac patients, comparing it with the non-celiac population. Additionally, we aimed to assess whether socio-demographic or CD-related factors are associated with a greater possibility of ED.

## METHODS

### Ethical issues

This is an analytical cross-sectional study.

The study was approved by the Research Ethics Committee (CEP) under number 4770592. All volunteer research participants signed an informed consent form agreeing to participate.

### Samples

The participants, self-declared as diagnosed with CD, were invited through support groups for celiac patients, whether belonging to celiac associations pages on Facebook, Instagram, and websites of associations that support celiac patients such as Acelpar (Associação de Celíacos do Paraná) and Fenacelbra (Federação Nacional das Associações de Celíacos do Brasil). Data were collected via online questionnaire from August 2021 to April 2022.

For the healthy control group, the questionnaire was sent by WhatsApp groups, e-mail, or Instagram. Participants in the control group declared that they did not have any chronic disease, especially DC. The two groups of participants were matched by sex and age.

For both groups, duplicate, inconsistent, and incomplete responses were excluded.

### Data collection

An online questionnaire was used in Google Forms format, which was configured in three sections. Participants in the control group only answered questions in sections 1–3.

*Section 1*: Demographic data: sex, age, schooling or level of education, and ethnicity.

*Section 2*: Data on CD diagnosis, symptoms; which difficulties related to GFD; and whether the GFD had professional guidance. Family history of CD, questions about the patient's weight, and depressive symptoms.

*Section 3:* Validated questionnaire Eating Attitudes Test-26 (EAT-26) in Portuguese. The EAT-26 has been used widely to measure the cognitive and behavioral symptoms of disordered eating in clinical and general population comprising males and females^12^, and it had 26 questions in the form of a Likert scale of points in which each answer has a value. The score is calculated from the sum of answers for each item, ranging from 0 to 78 points. Scores greater than 21 points are considered indicative of risky eating behavior, analyzing variables of bulimia, weight, body image, and psychological symptoms. EAT-26 is considered a reliable and valid instrument^12^.

### Statistical analysis

Statistical analyses were performed using the Graph Pad Prism 7.0 program. The Shapiro-Wilk test was applied to assess data normality. Continuous variables were expressed as mean and standard deviation or median and interquartile range (IQR) and compared using the non-parametric t-test or Mann-Whitney U test, as appropriate. Categorical variables were expressed as percentages and compared using Fisher's exact test or chi-square test, as appropriate. p<0.05 were considered statistically significant.

## RESULTS

A total of 835 responses were collected, of which 94 were excluded. Thus, 741 participants were studied, with 484 from the celiac group and 257 from the control group.

### Demographic data

The analysis of the sociodemographic data is available in Table 1. The average age of the studied patients was 38.1±3.8 years and that of the control group was 34.1±5.2 years.

**Table 1:** Demographic data of celiac patients and healthy controls.

Celiac patients (n=484)			Controls (n=257)	p
Age (years)	<18	0	0	0.31
	18–20	12 (2.4%)	17 (6.6%)	
	21–30	132 (27.2%)	114 (44.3%)	
	31–40	161 (33.2%)	41 (15.9%)	
	41–50	108 (22.35)	37 (14.3%)	
	51–60	51 (10.5%)	34 (13.2%)	
	>60	20 (4.1%)	14 (5.4%)	
Gender	Female	458 (94.6%)	213 (82.8%)	0.43
	Male	26 (5.4%)	44 (17.1%)	0.002
Schooling	Primary education	5 (1%)	2 (0.7%)	0.152
	High school	61 (12.7%)	41 (16.2%)	
	Incomplete high school	71 (14.6%)	100 (38.9%)	
	Graduation	134(27.6%)	61 (23.7%)	
	Postgraduation	217 (44.8%)	55 (21.4%)	
Ethnicity	Yellow	10 (2%)	13 (5%)	0.66
	White	408 (84.2%)	216 (84%)	
	Afrodescendants	66 (13.6%)	28 (10.9%)	

It was observed that there was no difference in the number of women who answered the questionnaire between the groups, as well as in schooling/education and ethnicity. However, the number of male respondents in the control group was significantly higher (p=0.002).

### Data about celiac disease

These questions were answered only by celiac patients and the data are available in Table 2. In the sample, 32.8% (159/484) had a family member with CD. Among them, 258 (53.3%) were satisfied with their weight. Regarding the diet, 86.1% (417/484) of the participants declared adherence to the GFD. Social networks were the most cited sources of information. Among those who complied with the GFD, 94% reported improvement in symptoms. Regarding feelings about having CD, 286 (59%) said they lived well with the disease, 155 (32%) declared difficulties, and 42 (8.6%) had many difficulties in living with CD.

**Table 2:** Clinical data of celiac patients studied (n=484).

		n (%)
Has a family member with CD? (n=484)	Yes	159 (32.8)
	No	323 (66.7)
Are you happy with your weight?	Yes	258 (53.3)
	No	226 (46.6)
Consume food with gluten?	>1x/week	8 (16.5)
	1x/week	21 (4.2)
	1x/month	30 (6.1)
	Eat without restrictions	6 (1.2)
	Never	417 (86.1)
How to guide yourself on the diet?	Instagram	97 (20.2)
	Sites	121 (25.1)
	Books	23 (4.7)
	Other	243 (50.2)
Improved symptoms with diet?	Yes	455 (94.4)
	No	17 (5.6)
How does it feel to have CD?	Live well	287 (59.0)
	I have difficulties	155 (32.0)
	I have many difficulties	42 (8.6)
Has a diagnosis of depression?	Yes	120 (24.7)
	No	362 (74.7)
Get treatment for depression?	Yes	116 (23.9)
	No	366 (75.6)
Has a diagnosis of eating disorders?	Anorexia	11 (2.2)
	Bulimia	12 (2.4)
	Binge eating disorder	11 (2.2)
	No	443 (91.5)
	Did not answer	7 (1.4)

Regarding the diagnosis of mental disorders, 120/484 (24.7%) had a diagnosis of depression, and 116 (23.9%) were treated for depression. Among participants with DC, 11 (2.2%) reported having anorexia, 12 (2.4%) bulimia, and 11 (2.2%) BED.

**Figure 1:**
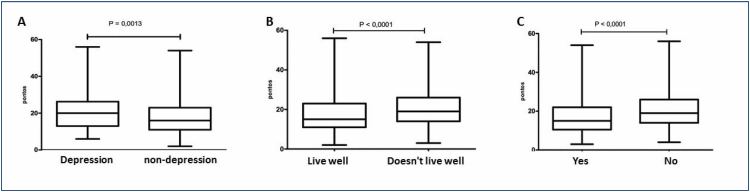
(A) Eating Attitudes Test-26 scores in celiac patients with and without depression. (B) Eating Attitudes Test-26 scores in patients who live well or not with celiac disease. (C) Eating Attitudes Test-26 scores in celiac patients who are or are not happy with their weight.

Among the 118 (24.3%) patients with CD who reported of having depression, the EAT score had an average of 20 points (IQR=13–26), and in the 362 (74.7%) who said they did not have depression, the average was 16 points (IQR=11–23; p=0.0013), as can be seen in Figure 1a. Among the 285/484 (58.8%) participants with CD who reported living well with the disease, the EAT scores had a median of 15 points (IQR=11–23). Among the 197 (38.6%) who reported not living well with DC, the median was 19 points (IQR=14–26), which was significantly higher (p<0.0001), as shown in Figure 1b. Of the participants with CD, 225 (46.4%) reported that they were not satisfied with their weight and had EAT scores with an average of 19 points (IQR=14–26). Among the 257 (53%) who reported being happy with their weight, an average of 15 points was observed (IQR=10–22; p<0.0001), as shown in Figure 1c.

### Data about Eating Attitudes Test-26

The average EAT score in patients with CD was 17 (IQR=12–25), and that of the control group was 18 (IQR=11–26; p=0.32). According to the classification of the scores obtained in the EAT-26 questionnaire, among celiac patients, 166/484 (34.2%) were at risk for developing ED, of which 157/458 (34.3%) were women. In celiac men, 9/26 (34.5%) were at risk for developing ED (p=0.35). In the comparison group, 97/357 (37.7%) people were at risk, with 86/213 (40.3%) being women. Of the men's group, 11/44 (25%) were found to be at some risk for developing ED (p=0.22). Schooling and age did not significantly influence EAT scores in the study sample (p=0.43 and p=0.38, respectively).

Comparing the two groups, celiac and control, no significant difference was observed between the number of individuals at risk for developing ED (p=0.39) and number of those who were not. Additionally, there was no difference between celiac women and controls (p=0.14) and between men in the celiac and control groups (p=0.42).

Among 34 celiac patients who said they had a diagnosis of ED, 23 (67.8%) had scores above 21 points. These data were not evaluated in controls, as having a diagnosed ED was an exclusion criterion.

## DISCUSSION

Although CD and ED are topics much discussed separately in the literature, there are few studies that associate these two diseases. Initially, the authors had the hypothesis that the dietary restriction imposed on celiac patients could increase the risk for ED. There are no reports on this topic in the Brazilian population. The way the investigation was carried out (online) provided a large sample (484 celiac patients) and the results did not demonstrate a greater risk for the celiac group when compared with the control group. On the contrary, our data indicated, in both studied groups, that about one-third of the participants had a risk score for developing ED.

Most of the participants in this study were females and educated. In general, women have better adherence (>80%) to online questionnaires compared with men^13^. Additionally, DC affects a greater number of women^8^. There were no significant differences in the answers between genders in both groups in the topics studied.

Two articles that correlate both diseases were found in the European literature. The parameter used in their risk assessment method was the EAT-26 questionnaire, which has a sensitivity of 82% and is efficient for screening ED^14^. However, they showed different results. A study carried out in Italy included adult patients with untreated CD and a possible association of this group with behaviors suggestive of ED. The data presented indicated that the behavior of ED was higher in patients with CD and in women^12^. Our study obtained a different result, showing that there was no association between ED and CD. Another study in Spain obtained a similar result to our data, with no significant difference between controls and celiac patients^9^.

It is suggested that patients with CD have a more restricted diet, leading them to have more guidance on nutrition and health care.

From the responses obtained on the questionnaire directed only at patients with CD, 94% of them improved their symptoms with the GFD. Probably, with the improvement of gastrointestinal symptoms and disease control, these patients had a slight weight gain. When asked if the celiac participant was satisfied with their weight, almost half of the sample was not happy with their body weight. In that same sample, more than 90% denied any type of ED. However, according to the EAT assessment, 34.2% are at risk for developing one of these disorders. It is suggested that probably these patients are underdiagnosed.

For participants with CD diagnosed with depression, difficulties in living with CD and being dissatisfied with their weight are significantly associated with higher EAT scores. Therefore, they are at greater risk for developing ED. Other studies show that, although patients with depression face symptoms of ED, they do not meet the DSM criteria to be diagnosed as such. However, patients with BED are associated with a higher risk of suicide^15^.

Furthermore, it can be observed that 67.8% of patients with CD who claimed to have a diagnosis of ED showed a high score on the EAT-26. It is possible that they are not being adequately treated for AT.

This study has some limitations related to its cross-sectional design and the data collection method used. The survey was conducted online, without access to medical records of patients with CD, which may lead to inaccuracies. However, we sought to disseminate the survey questionnaire also in groups of patients previously diagnosed with CD, in order to avoid the responses of people without the disease. The high-risk index observed in both groups may be associated with the fact that people who are already at risk for developing ED may be more interested in answering a questionnaire on the subject, creating a selection bias. In addition, disclosure was mainly via social networks and some patients did not have access to the form. On the contrary, the online survey allows for the participation of a greater number of people and does not cause embarrassment due to its anonymous character, making the answers more reliable.

In conclusion, there was no greater risk for celiac patients to develop ED compared with the control group. However, for both groups, attention was drawn to the high frequency of people at risk for having one of the types of ED, given that one-third of respondents are at risk in both groups. Depression, difficulties in living with CD, and being dissatisfied with one's own weight are significantly associated with higher EAT scores, suggesting a greater chance of developing ED.
